# Polymorphisms in the Toll-like receptor 3 (*TLR3)* gene are associated with the natural course of hepatitis B virus infection in Caucasian population

**DOI:** 10.1038/s41598-018-31065-6

**Published:** 2018-08-24

**Authors:** Janett Fischer, Eleni Koukoulioti, Eckart Schott, Balazs Fülöp, Renate Heyne, Thomas Berg, Florian van Bömmel

**Affiliations:** 1 Department of Gastroenterology and Rheumatology, Section of Hepatology, University Hospital Leipzig, Leipzig, Germany; 2 Department of Gastroenterology, Hepatology and Diabetology, Internal Medicine II, HELIOS Hospital Emil von Behring, Berlin, Germany; 3 Department of Internal Medicine and Gastroenterology, HELIOS Hospital Berlin-Buch, Berlin, Germany; 4 Liver and Study Center Checkpoint, Berlin, Germany

## Abstract

Innate immunity can induce spontaneous hepatitis B surface antigen (HBsAg) seroclearance (SC) of hepatitis B virus (HBV) infection or transition towards an inactive carrier state. Toll-like receptor (TLR) 3 signalling has been linked to these processes. Alterations in the *TLR3* gene might impair immune responses against HBV. In our study, we analysed the impact of the *TLR3* polymorphisms rs3775291 and rs5743305 on the natural course of HBV infection. In this retrospective study, a Caucasian cohort of 621 patients with chronic HBV infection (CHB), 239 individuals with spontaneous HBsAg SC, and 254 healthy controls were enrolled. In the CHB group, 49% of patients were inactive carriers, and 17% were HBeAg-positive. The *TLR3* rs3775291 A allele was associated with a reduced likelihood of spontaneous HBsAg SC and HBeAg SC, and an increased risk of developing chronic hepatitis B. In haplotype analysis, the haplotype including both risk variants rs3775291A and rs5743305A had the lowest likelihood of HBsAg SC. Further research in larger cohorts and functional analyses are needed to shed light on the impact of TLR3 signalling.

## Introduction

The risk of developing chronic hepatitis B virus (HBV) infection and its complications correlate with the disease stage, which reflects the degree of immune control. Thus, liver cirrhosis and hepatocellular carcinoma (HCC) occur more often in the active phase of the disease, but their prevalence is reduced if hepatitis B e antigen (HBeAg) seroconversion, inactive carrier (IC) state and hepatitis B surface antigen (HBsAg) loss occurs^1^. The mechanisms underlying this immune control over the HBV infection have not been fully explained. It is, however, known that adaptive immune responses are required to resolve the infection, especially HBV specific T cells^2,3^. In contrast, the role of innate immunity in the control of HBV infections remains controversial. Recent studies showed the effect of Toll-like receptors (TLR) on HBV infection by initiating antiviral responses and stimulating adaptive immune responses^4–6^. Furthermore, TLR-mediated immune responses are shown to inhibit HBV replication in hepatocytes and animal models^7–9^. Interactions between TLR2, TLR3, TLR4, TLR7 and TLR9 and HBV have been reported previously^5,6,10^.

TLR3 detects double-stranded (ds) RNA from viruses, endogenous dsRNA and synthetic polyinosinic:polyribocytidylic acid (poly I:C). TLR3 signalling leads to activation of transcription factors such as interferon-regulatory factor-3 (IRF3) and nuclear factor (NF)-кB and induces the production of interferon-β and inflammatory cytokines^11^. The receptor is expressed in hepatocytes as well as in macrophages, natural killer (NK) cells and biliary epithelial cells, and is located in the plasma membrane or acidic endosomes^5^. Macrophages and NK cells are essential for immune recognition and virus eradication in innate and early adaptive immune responses against HBV^12,13^. Furthermore, TLR3 can activate hepatic non-parenchymal cells (NPCs) such as Kupffer cells, liver sinusoidal endothelial cells to produce interferon-β during HBV infection^8^.

Single nucleotide polymorphisms (SNPs) within the *TLR3* gene may cause changes in the protein or gene expression, which affects the function and efficacy of signal transduction and thus an altered immune response. In previous reports, *TLR3* polymorphisms rs1879026, rs3775296, rs3775291, rs5743305 have been associated with the outcome of hepatitis C virus (HCV) and HBV infection, and the development of consequential liver cirrhosis and HCC primarily in Asian populations^14^. Similarly, Al-Qahtani *et al*. showed a significant effect of the haplotype GCGA, composed of the four SNPs rs1879026, rs5743313, rs5743314, and rs5743315, on the susceptibility of HBV infection in persons from Saudi Arabia^15^. Huang *et al*. also identified the SNP rs3775290 as a protective factor against the development of chronic HBV infection and advanced stages of liver disease in the Chinese population^16^. Thus, evidence suggests that a link exists between TLR3 variants and immune control over HBV infections but to date, a clear association in a large Caucasian patient population is lacking.

This study investigates the presence of the *TLR3* SNPs rs3775291 and rs5743305 in a large multicentre cohort of patients with HBV infection and healthy controls of Caucasian ethnicity. We selected these SNPs as they have recently been found to represent new risk factors of HBV-related diseases in an Asian population^14^ and affect TLR3 signalling^17–19^. We aimed to assess the impact of these SNPs on the susceptibility of chronic HBV infection (CHB), spontaneous HBsAg seroclearance (SC) and the occurrence of different disease stages of CHB such as HBeAg SC and inactive carrier (IC) state.

## Results

### Patient characteristics

**Figure 1:**
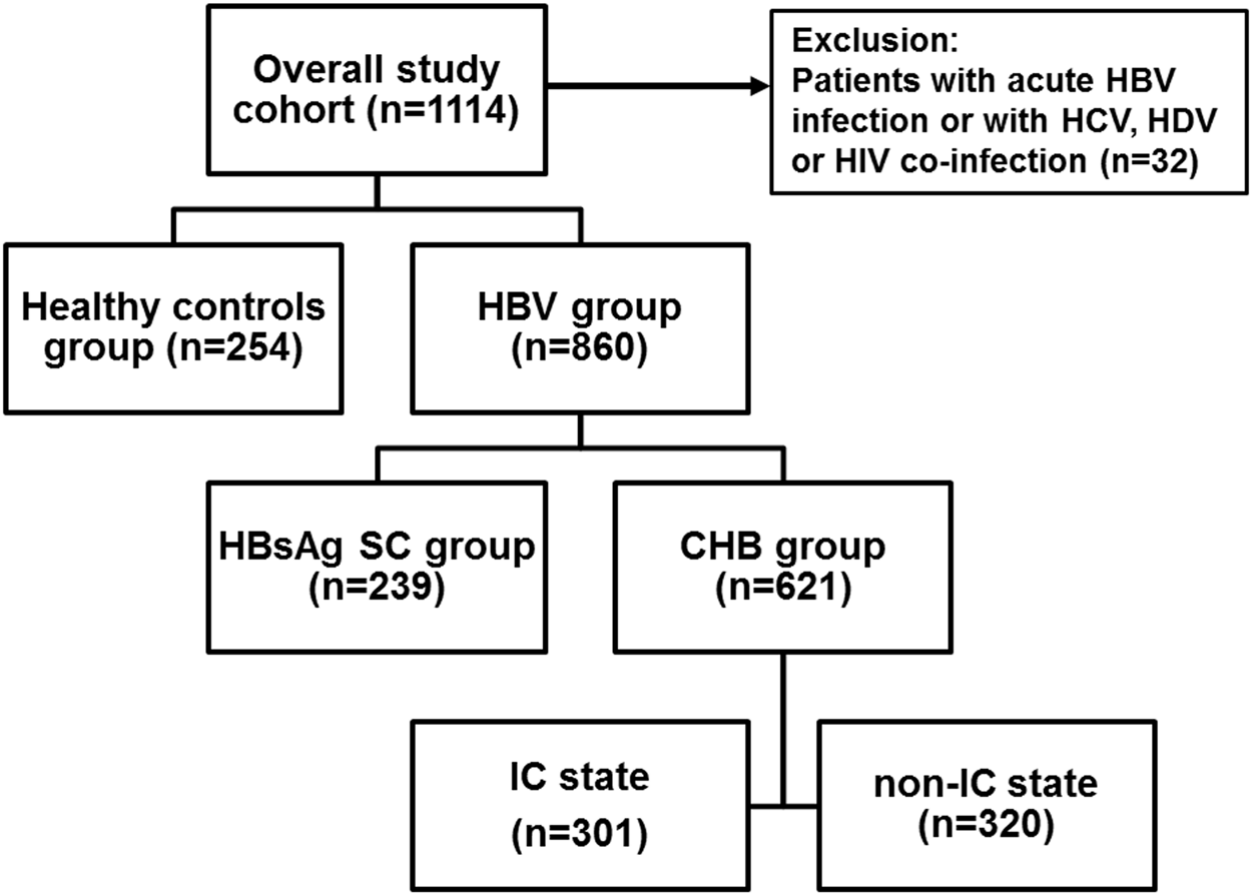
Overview of the investigated study population. The overall study population included healthy controls and the HBV group. Patients with acute HBV infection or HCV, HDV or HIV co-infection were excluded from the study. Chronic hepatitis B (CHB) was characterized by the presence of HBsAg and HBV DNA for more than six months and spontaneous HBsAg seroclearance (HBsAg SC) was defined by undetectable HBsAg and detectability of anti-HBs and total anti-HBc antibodies. The CHB group was further divided into an inactive carrier (IC) state and in a non-IC state according to the current European guidelines^1^.

The subdivision of the overall study cohort is presented in Fig. 1. Baseline characteristics of the study cohort (n = 1114) are shown in Table 1. Individuals in the CHB group were significantly younger (Man-Whitney U = 4830.5, p = 1.29 × 10^−22^) and included more females (χ^2^ = 4.72 p = 0.03) as compared to the group of patients with spontaneous HBsAg seroclearance (HBsAg SC) group. The HBsAg SC group had significantly more patients with liver cirrhosis (χ^2^ = 4.94 p = 0.031) and elevated alanine transaminase (ALT) levels (Mann-Whitney U = 52578.5, p = 0.002) than the CHB group. However, cirrhosis development and elevated ALT levels in the HBsAg SC patients was primarily caused by excessive alcohol consumption (44%), followed by idiopathic (32%) causes, autoimmune (12%) and non-alcoholic liver diseases (12%).

**Table 1:** Baseline characteristics of the (a) overall HBV cohort and the control group and (b) the CHB patients in inactive carrier (IC) state or non-IC.

Parameter	(a) HBV cohort			Control		(b) CHB					
	HBsAg SC (n = 239)	CHB (n = 621)	P-value	Controls (n = 254)	P-value^†^	IC state (n = 301)	non-IC state (n = 320)	P-value	HBeAg + (n = 103)	HBeAg- (n = 518)	P-value
Age (years)*	64.9 ± 14.3	53.6 ± 13.9	1.29 × 10^−22^	63.5 ± 2.8	3.10 × 10^−28^	52.4 ± 13.5	54.7 ± 14.3	0.088	53.4 ± 14.2	53.6 ± 13.8	0.675
Male gender	128 (53.6%)	383 (61.7%)	0.03	45 (17.7%)	5.62 × 10^−34^	164 (54.5%)	219 (68.4%)	0.0004	79 (76.7%)	304 (58.7%)	0.001
Inactive carriers	n.a.	301 (48.5%)		n.a.					4 (3.9%)**	297 (57.3%)	3.89 × 10^26^
HBeAg-positive	n.a.	103 (16.6%)		n.a.		4 (1.3%)**	99 (30.9%)	4.17 × 10^−27^			
Cirrhosis	50 (20.9%)	92 (14.8%)	0.031	n.a.		28 (9.3%)	63 (19.7%)	0.0003	22 (21.4%)	69 (13.3%)	0.046
Hepatocellular carcinoma	9 (3.8%)	41 (6.6%)	0.142	n.a.		10 (3.3%)	31 (9.7%)	0.002	8 (7.8%)	33 (6.5%)	0.663
Previous or current antiviral treatment	n.a.	352 (56.7%)		n.a.		87 (28.9%)	264 (82.5%)	2.76 × 10^−43^	99 (96.1%)	252 (48.5%)	7.77 × 10^−20^
HBV DNA (log_10_ IU/ml)*	n.a.	3.4 ± 2.4*		n.a.		2.0 ± 1.2	4.7 ± 2.5	2.15 × 10^−47^	6.04 ± 2.22	2.85 ± 2.04	7.15 × 10^29^
ALT (IU/L)*	69.3 ± 327.15	61.6 ± 140.1*	0.0002	n.a.		31.8 ± 23.3	89.9 ± 190.3	7.53 × 10^−19^	85.32 ± 107.4	56.95 ± 145.2	1.47 × 10^−9^

^†^p-value from the comparison of the CHB group with the controls, CHB: chronic hepatitis B, SC: seroclearance, *mean ± SD, n.a. not applicable. **HBeAg loss during observation time.

Comparisons of continuous variables were made using the Mann-Whitney U test. Categorical variables were compared with the Pearson’s χ^2^ test.

In the CHB group, more females developed an IC state (χ^2^ = 12.77 p = 0.0004) than males. And within the non-IC group, there were significantly more patients with liver cirrhosis (χ^2^ = 13.38 p = 0.0003) and fewer patients with HCC (χ^2^ = 10.19 p = 0.002). Moreover, baseline HBV DNA (Mann-Whitney U = 14864.0, p = 2.15 × 10^−47^) and ALT levels (Mann-Whitney U = 27722.5, p = 7.53 × 10^−19^) were significantly lower in the IC group.

### Prevalence of *TLR3* SNPs in individuals with and without HBV infection

**Figure 2:**
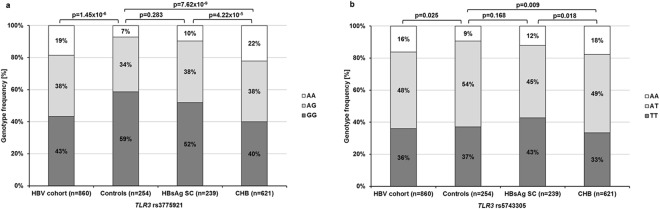
Genotype distributions of the *TLR3* SNPs rs3775291 (**a**) and rs5743305 (**b**) in the overall cohort and in the sub-groups. Frequencies were compared with Pearson’s χ^2^ test for categorical variables. The genotype distribution of both SNPs differed significantly between the study cohort and healthy controls and between the CHB and HBsAg SC groups (HBsAg: hepatitis B surface antigen, SC: seroclearance and CHB: chronic hepatitis B).

The genotype distributions of both *TLR3* SNPs rs3775291 and rs5743305 differed significantly between the healthy controls and the HBV cohort (Fig. 2). The A allele of rs3775291 (χ^2^ = 18.69 p = 1.69 × 10^−5^) and the AA genotype of rs5743305 (χ^2^ = 7.08 p = 0.008) were overrepresented in the HBV cohort.

When the HBV cohort was further divided into the HBsAg SC and CHB group, the differences in *TLR3* SNPs genotype distribution only remained significant between the controls and the CHB group (Fig. 2). Thus, univariate logistic regression analysis revealed a higher likelihood of CHB for the *TLR3* rs3775291 A allele (odds ratio [OR] = 2.13 [95% confidence interval [CI]: 1.59–2.87] p = 5.60 × 10^−7^) and the rs5743305 AA genotype (OR = 2.06 [95% CI: 1.29–3.30] p = 0.002). In adjusted multivariate logistic regression analysis, both SNPs remained significantly associated with CHB (rs3775291A: OR = 2.45 [95% CI: 1.69–3.48] p = 1.65 × 10^−6^ and rs5743305A: OR = 3.031 [95% CI: 1.74–5.29] p = 9.55 × 10^−6^). Furthermore, age-matched analysis revealed an increase in power regarding the association of the risk variants with CHB (rs3775291A: OR = 3.26 [95%CI: 1.96–5.42] p = 5.18 × 10^−6^ and rs5743305A: OR = 3.59 [95% CI: 1.80–7.18] p = 0.0003).

### Association of *TLR3* SNPs with spontaneous HBsAg SC of HBV infections

Genotype distributions of both *TLR3* SNPs differed significantly between the HBsAg SC and the CHB groups (Fig. 2). In univariate logistic regression analysis, the rs3775291 AA genotype was associated with a reduced likelihood of spontaneous HBsAg SC with an OR of 0.38 (95% CI: 0.24–0.60, p = 4.54 × 10^−5^) under a recessive model and the A allele (risk variant) with an OR of 0.62 (95% CI: 0.46–0.83, p = 0.002) under a dominant model; the *TLR3* rs5743305 A allele (risk variant) was associated with an OR of 0.67 (95% CI: 0.50–0.91, p = 0.011) under a dominant model, respectively (Table 2).

**Table 2:** Genotype distribution of the *TLR3* SNPs and the association with spontaneous HBsAg seroclearance (SC) using logistic regression analysis.

*TLR3*		CHB (n = 621)	HBsAg SC (n = 239)	Unadjusted OR (CI 95%)	P-value	Adjusted OR (CI 95%)	P-value
rs3775291	GG	248 (39.1%)	124 (51.9%)				
	AG	236 (38.0%)	92 (38.5%)				
	AA	137 (22.1%)	23 (9.6%)				
	MAF	0.41	0.29				
	AA/AG vs. GG			0.62 [0.46–0.83]	0.002	0.58 [0.42–0.80]	0.001
	AA vs. AG/GG			0.38 [0.24–0.60]	4.54 × 10^−5^		
rs5743305	TT	207 (33.3%)	102 (42.7%)				
	AT	304 (49.0%)	108 (45.2%)				
	AA	110 (17.7%)	29 (12.1%)				
	MAF	0.42	0.35				
	AA/AT vs. TT			0.67 [0.50–0.91]	0.011	0.66 [0.48–0.93]	0.016
	AA vs. AT/TT			0.64 [0.41–0.99]	0.048		

CHB: chronic hepatitis B, SC: seroclearance, OR: odds ratio, CI: confidence interval.

Since age, gender and patient origin are important risk factors for the development of chronic HBV infection, we performed adjusted multivariate logistic regression analysis and confirmed the strength of the association of both risk variants of *TLR3* rs3775291 (OR = 0.58 [95% CI: 0.42–0.80] p = 0.001) and rs5743305 (OR = 0.66 [95% CI: 0.48–0.93] p = 0.016) with HBsAg SC (Table 2). An increase in power was detected using age-matched groups (rs3775291A: OR = 0.47 [95%CI: 0.30–0.72] p = 0.001 and rs5743305A: OR = 0.55 [95% CI: 0.35–0.86] p = 0.008).

### Haplotype analysis of *TLR3* rs5743305 and rs3775291

**Figure 3:**
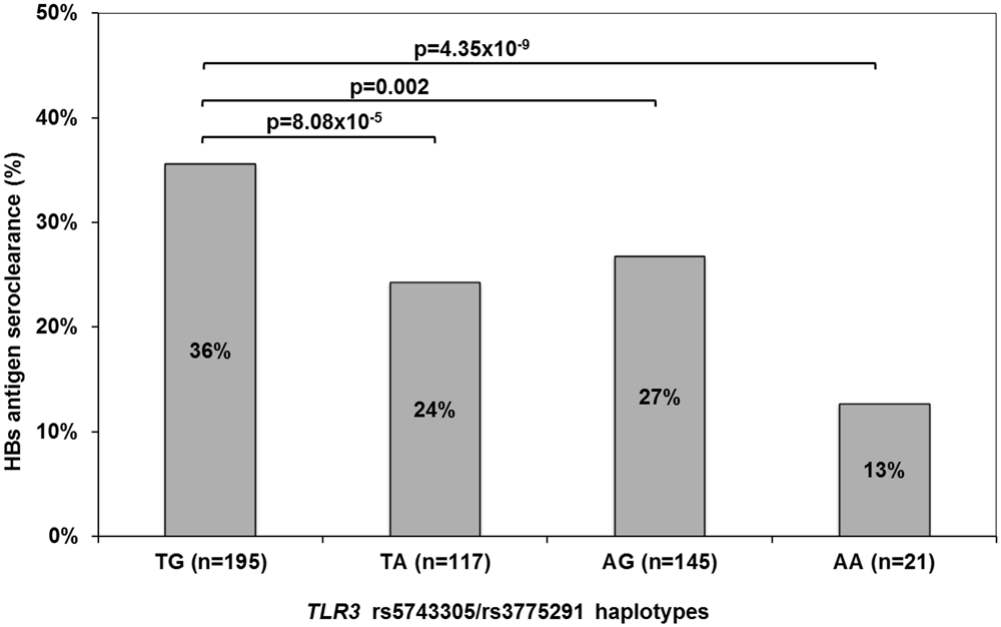
HBsAg seroclearance rates of the *TLR3* rs5743305/rs3775291 haplotypes.

In our cohort, the *TLR3* SNPs rs5743305 and rs3775291 are in weak linkage disequilibrium (LD) (D’ = 0.058, r^2^ = 0.001). Therefore, four haplotypes exist: rs5743305T/rs3775921G (36.4%), rs5743305T/rs3775921A (25.9%), rs5743305A/rs3775921G (23.4%) and rs5743305A/rs3775921A (14.2%). Carriers of at least one A allele of the *TLR3* SNPs had lower chances of HBsAg SC than carriers of the wild-type genotypes, for example, 24% TA vs. 37% TG χ^2^ = 15.54 p = 8.08 × 10^−5^ (Fig. 3). The lowest likelihood of HBsAg SC was assessed for the AA haplotype comprising both risk variants with an OR of 0.26 (95% CI: 0.16–0.43, p = 8.51 × 10^−8^) compared to the TG haplotype (Table 3).

**Table 3:** Haplotypes of TLR3 rs5743305/rs3775291 associated with spontaneous HBsAg seroclearance in the study cohort using logistic regression analysis.

*TL3* rs5743305/rs3775291			
Haplotype	Frequency	OR [95% CI]	p-value
TG	0.364	REF	
TA	0.259	0.58 [0.44–0.76]	8.75 × 10^−5^
AG	0.234	0.66 [0.51–0.86]	0.002
AA	0.142	0.26 [0.16–0.43]	8.51 × 10^−8^

OR = odds ratio, CI = confidence interval, REF = reference.

### Association of the *TLR3* SNPs with hepatitis B disease stages

We examined the association of both *TLR3* SNPs with the disease stages of chronic HBV infection, specified through the following analyses: 1) HBV DNA levels, 2) ALT levels; 3) the IC state vs. non-IC state; 4) HBeAg-negative vs. HBeAg-positive CHB; and the presence or absence of 5) cirrhosis and 6) HCC across the CHB cohort.

Patients carrying the *TLR3* rs3775291 or rs5743305 risk variants had higher HBV DNA and ALT levels than carriers of the GG or TT genotype (rs3775291: HBV DNA log_10_ IU/mL: p = 8.55 × 10^−8^ and ALT IU/mL: p = 0.001; rs5743305: p = 0.006 and p = 0.017, respectively) using Mann-Whitney U test.

**Figure 4:**
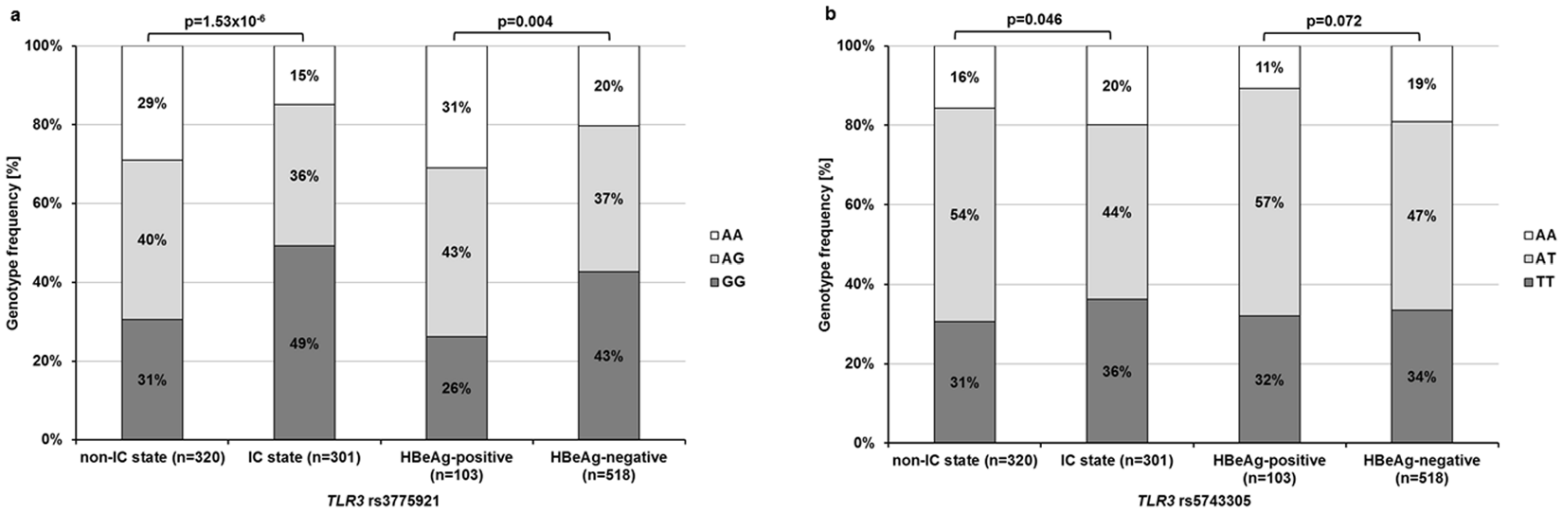
Genotype distributions of the *TLR3* SNPs rs3775291 (**a**) and rs5743305 (**b**) in the CHB group. Frequencies were compared with Pearson’s χ^2^ test for categorical variables. The genotype distributions of both SNPs significantly differed between patients with non-IC and IC states. Only the SNP rs3775291 was significantly different between HBeAg-positive and HBeAg-negative patients. IC: inactive carrier.

The genotype distribution of *TLR3* rs3775291 was significantly different between all groups, and the distribution of rs5743305 only between the IC and non-IC groups, respectively (Fig. 4). Univariate logistic regression analysis revealed a significant association of the *TLR3* rs3775291 risk variant with a higher likelihood of development of active chronic hepatitis B (OR = 2.13 [95% CI: 1.54–2.95] p = 6.00 × 10^−6^) and with HBeAg positivity (OR = 2.10 [95% CI: 1.31–3.36] p = 0.002). By using Bonferroni correction, the rs3775291 risk variant remained significantly associated with non-IC, with an OR of 2.16 (95% CI: 1.55–3.01, p = 5.50 × 10^−6^) and HBeAg presence, with an OR of 2.06 (95% CI: 1.28–3.33, p = 0.003). For both SNPs, there was neither an association with the presence of cirrhosis nor the development of HCC.

## Discussion

In this large multicentre Caucasian population study, we showed for the first time a strong association of the common SNP rs3775291 in the *TLR3* gene with different stages of immune control over chronic HBV infections and with spontaneous HBsAg SC.

To date, the functional relevance of SNPs in the *TLR3* gene is not entirely known. The substitution of G to A at position rs3775291 leads to an amino acid change from leucine to phenylalanine at position 412 of the protein. This alteration reduces the localisation of the soluble ectodomain and the dimerization of TLR3 at membranes, resulting in its decreased binding capacity to dsRNA and a lower signalling activity compared to the wild-type^17,18^. The polymorphism rs5743305 is located in the promotor region of the *TLR3* gene and is suggested to influence transcriptional activity. However, Askar and colleagues detected no impaired *TLR3* gene expression in peripheral blood mononuclear cells (PMBCs) of heterozygous or homozygous rs5743305 variants compared to wild-type^19^. Although their effect on transcriptional activity is not completely clear, both SNPs are shown to be associated with low humoral and cellular response to measles vaccination. For example, the heterozygous variants of both SNPs were associated with lower levels of measles-specific antibodies (wild-type vs. variant: rs3775291 p = 0.02, rs5743305 p = 0.004), and the heterozygous variant of rs5743305 showed a lower lymphoproliferative response compared to the wild-type (p = 0.003)^20^.

In our study, the risk variants of rs3775291 and rs5743305 were more prevalent in CHB group compared to healthy controls (Fig. 2), a difference that may have been influenced by a lower risk exposure of the control group. However, those patients who achieved spontaneous HBsAg SC showed a similar distribution of the risk variants as controls. Interestingly, the difference between the HBsAg SC and the CHB groups was similar to the difference between the CHB group and control, suggesting an influence of both risk variants on the immune control of HBV infections.

Another marker of immune control over HBV infections is the occurrence of HBeAg SC. In our study, the incidence of the GG genotype of *TLR3* rs3775291 was higher in patients who had achieved HBeAg SC in comparison to HBeAg-positive patients. The AA genotype was more frequent in HBeAg-positive patients, which further supports an association of the rs3775291 variants with an altered innate immune response during HBV infection. Similar to the rs3775291 SNP, the risk variant of rs5743305 was also associated with disease stages in CHB. However, the associations were not independent of the rs3775291 SNP, suggesting a possible relationship between both *TLR3* polymorphisms.

Because haplotypes are considered to be more informative than single-locus analyses with regard to associations with complex diseases^21^, we performed haplotype analyses for *TLR3* rs3775291 and rs5743305. We detected an additive effect of both SNPs. The presence of at least one risk allele reduced the likelihood of HBsAg SC rates by ~40% compared to wild-type, and the presence of two risk alleles up to 75%, respectively.

Our observation regarding the association of *TLR3* risk variants with HBsAg SC contradicts findings of previous studies^16,22–24^. Sa *et al*. observed no difference of both SNPs between chronically infected patients (n = 35) and healthy subjects (n = 299) from Brazil^23^. In Asian population, HBV is mainly passed by perinatal transmission, whereas HBV in Caucasians is primarily adult-acquired by exposure to infected blood and various body fluids^25^. Therefore, both rates of HBsAg SC and epidemiology of CHB differ between the populations. Nevertheless, this study and other previous reports show a link between *TLR3* SNPs and HBV susceptibility^16,23,24^, and our study is the first to show an association of the risk variants of rs3775291 and rs5743305 with decreased HBsAg SC in Caucasian patients.

Other *TLR3* variants that have not been analysed in our study may also play a role in the immune control of HBV infection. Interestingly, Goktas *et al*.^26^ reported that in Turkish patients with active CHB there was a higher prevalence of the CC genotype of the *TLR3* SNP rs3775290, which is located close to rs3775291. They suggest that the wild-type might lead to a stronger immune response against HBV. Conversely, in our study, the *TLR3* rs3775291 risk variant presented higher baseline HBV DNA and ALT levels as well as an association with active CHB. This might be due to the impaired functional activity of TLR3 with subsequent decreased HBV recognition and increased HBV propagation in the liver.

We were unable to detect any association between *TLR3* risk variants and liver cirrhosis or HCC, which is contrary to findings by Li *et al*.^27^ and Chen *et al*.^22^ in Asian patients. Their studies suggest that the *TLR3* SNP rs3775291 is a novel risk factor for HBV-related HCC. The lack of an association with HCC might be due to the limited number of patients with advanced liver disease or HCC in our cohort, or the different ethnic background. Therefore, further investigations are needed in large cohorts of patients with HBV-related HCC.

One limitation of our study is the yet unproven functional relevance of *TLR3* in HBV infections. Although HBV is known to be a “stealth” virus which does not trigger an interferon response in infected hepatocytes^28–30^, innate immunity of liver-resident macrophages, Kupffer cells and NPCs can be activated^4,8,31,32^. The HBV replication phase with increased production, assembly and release of HBV particles from hepatocytes results is a higher local exposure to these immune cells^30^. Future studies need to confirm if TLR3 signalling can be activated by the internalisation of the immature RNA-containing virions, which are being secreted during HBV production^33^, or if other mechanisms are involved. However, recent investigations show that stimulation of TLRs with exogenous ligands improves immune responses against HBV^5,7–9,34–37^, and can induce the production of inflammatory cytokines and chemokines in chronically infected HBV patients^36^. Furthermore, TLR agonists also activate cytotoxic T lymphocyte responses and inhibit HBV propagation^7–9,37^. Triggering of TLR-mediated pathways will become critical in approaching host factor-targeted treatment strategies to cure HBV infection^35,38^. Thus, polymorphism in genes of key components of the TLR-signalling pathways might also affect individual therapy outcome.

In conclusion, the current study shows for the first time that the *TLR3* gene GG genotype of the SNPs rs3775291 and the TT genotype of rs5743305 are associated with HBsAg SC and IC state, and the GG genotype of rs3775291 is linked to HBeAg SC in Caucasians. Thus, TLR3 may represent an important factor in immune control over HBV infection. Nonetheless, large population-based studies in HBV populations with different genetic backgrounds, as well as testing for additional TLR3 variants and functional analyses, are needed to understand the effect of genetic variations in the complex mechanisms on immune control during the different phases of HBV infection.

## Patients and Methods

### Patients

A total of 1114 patients with HBV infection and healthy controls of Caucasian origin from two academic hepatology centres in Germany (Section of Hepatology, University Hospital of Leipzig, Germany and Department of Hepatology and Gastroenterology, University Hospital Charité, Berlin, Germany) and one primary health provider (Liver and Study Center Checkpoint, Berlin, Germany) were enrolled onto the study between 2003 and 2015. Patients with acute HBV infection or HCV, HDV or HIV co-infection were excluded from the study.

The overall study population included: a control group of 254 unrelated healthy blood donors (all with undetectable HBsAg and total anti-HBc antibodies) and 860 patients in the HBV group, which included the CHB group (n = 621) with the presence of HBsAg and HBV DNA for more than six months, and the HBsAg SC group (n = 239 patients) with spontaneous HBsAg SC, defined by undetectable HBsAg and detectability of anti-HBs and total anti-HBc antibodies. The patients in the CHB group were further divided into those in an IC state (n = 301, HBeAg-negative and HBV DNA levels <2,000 IU/mL, persistently normal serum ALT levels) and those with non-IC state (n = 320, HBV DNA level >2,000 IU/mL or elevated serum ALT levels in the absence of secondary liver disease), according to the current European guidelines^1^ (Fig. 1). Caucasian was defined as patients descended from Northern/Central or Eastern Europe (n = 859), the Mediterranean region (Turkey, Greece or Italy, n = 229) or the Middle East (Iran, Afghanistan, n = 26).

Since HBV infection often presents entirely asymptomatic during the acute phase, and the infection goes unrecognised in a large proportion of affected patients^39^, the age at first infection and the route of transmission were not available for numerous patients, especially for those who spontaneously cleared the virus. Moreover, the chronic patients were from academic liver centres, and selection bias cannot be excluded.

Liver cirrhosis was diagnosed by radiological evidence and/or a liver biopsy. The diagnosis of HCC was based on histological examination of tumour tissue or evidence on imaging^40^.

### Genotyping

The DNA samples were analysed from whole blood samples stored at −20 °C for the *TLR3* SNPs rs3775291 and rs5743305. DNA was extracted from whole blood samples with an extraction kit from QIAGEN (Hilden, Germany). Genotyping was performed by real-time polymerase chain reaction (PCR) and melting curve analysis in a Light Cycler 480 System (Roche) using fluorescence resonance energy transfer (FRET) probes (TIB MOLBIOL, Berlin, Germany). PCR conditions and primer/probes sequences are shown in the Supplementary Material. Sequencing was performed with BigDye Terminator and a capillary sequencer from Applied Biosystems (Darmstadt, Germany).

### Statistics

Statistical analyses of epidemiological associations were performed using SPSS software (SPSS Inc., version 24.0, Chicago, IL, USA). The genotype distributions of the two SNPs were tested for deviations from the HWE^41^ using the DeFinetti program with a cut-off p-value of 0.01.

Comparisons of the distributions of demographical characteristics between the different groups were made using the Mann-Whitney U test for continuous variables (each when adequate) and the Pearson’s χ^2^ test and Fisher’s exact test for categorical variables. Univariate and multivariate logistic regression analyses were performed to determine the association between the SNPs and the disease status under dominant and recessive genetic models.

All tests were two-sided and p-values less than 0.05 were considered statistically significant. The OR and the 95% CI were calculated. We aimed to estimate both the recessive and additive effects of the SNPs. Structure of LD was analysed with Haploview 4.2 (Broad Institute, Cambridge, USA) by using the expectation-maximization (EM) algorithm. The LD present between the single SNPs. D’ varies from 0 (complete equilibrium) to 1 (complete disequilibrium). R^2^ shows the correlation between SNPs. When r^2^ = 1, two SNPs are in perfect LD, and allelic frequencies are identical for both SNPs^41^.

### Ethics statement

The study was approved by the Ethics Committees of Medical Research of the University of Leipzig and Berlin in accordance with the Declaration of Helsinki from 1975 (revision 2013) and the International Conference on Harmonization/Committee for Proprietary Medicinal Products “Good Clinical Practice” guidelines. All patients provided written informed consent.

## Electronic supplementary material

Supplementary Dataset 1

## Data Availability

The datasets generated and/or analysed during the current study are available in the public repository of the University Leipzig under, http://ul.qucosa.de/.
