# Supplementary material for: Polymorphisms in the Toll-like receptor 3 (TLR3) gene are associated with the natural course of hepatitis B virus infection in Caucasian population
Source: Sci Rep. 2018 Aug 24;8:12737. doi: 10.1038/s41598-018-31065-6 (PMC6109130; doi:10.1038/s41598-018-31065-6)
Supplement: Supplementary file 1 — Supplementary Dataset 1 [file 41598_2018_31065_MOESM1_ESM.docx]

**Supplementary Information:**

**Polymorphisms in the Toll-like receptor 3 (*TLR3)* gene are associated with the natural course of hepatitis B virus infection in Caucasian population**

Janett Fischer^1*^, Eleni Koukoulioti^1^, Eckart Schott^2^, Balazs Fülöp^3^, Renate Heyne^4^, Thomas Berg^1^, Florian van Bömmel^1^

**Correspondence to:** Janett Fischer, Department of Gastroenterology and Rheumatology, Section of Hepatology, Liebigstr. 21, 04103 Leipzig, Germany, janett.fischer@medizin.uni-leipzig.de

**Polymerase chain reaction conditions**

Polymerase chain reaction (PCR) amplification and melting curve analysis was carried out in a total volume of 10 µl containing 5.0 µl Light Cycler 480 Probes Master with 6.4 mM MgCl_2_, 3.6 µl PCR-grade H_2_O, 0.5 mM each primer, 0.1 mM sensor probe and 0.1 mM anchor probe. PCR conditions were: initial denaturation at 95°C for 12 min; followed by 45 cycles of denaturation at 95°C for 20s; annealing at 60°C for 40s and extension at 72°C for 1.5 min. Melting curves were detected after denaturation at 95°C for 5s; holding the sample at 40°C for 20s, and then heating the sample to 80°C with a ramp rate of 0.14°C/s and continuous fluorescence acquisition. Sequencing was used to confirm results from the melt curve analysis, which have failed clear genotyping.

Table 1: Sequences of primer and probes for melting curve analysis and sequencing.

| TLR3 SNPs | Primer/probes | Sequence (5’-3’) |
| --- | --- | --- |
| rs3775291 | sense  antisense  sensor  anchor | GCAAAGGAGTTCCTAGTCAGC  GGAAGATAATGATATTCCAGGCAT  TCTTGGTTAGGTTGA**A**TATGTGTAAG-FL  LC610-GAGAATGAGCAAGTGATACAAATGTTTCAT-Ph |
| **rs5743305** | sense  antisense  sensor  anchor | GTCACGTACTCAGGACATCTG  CTGGACGGCTGGGACATG  AGCTAG**T**CCGCTTTATAGTTAC-FL  LC610-GGGCTCAGGGCTACATGTCTGTC-ph |
| FL= Fluorescein, LC=Light Cycler; Ph=phosphate, SNP=single nucleotide polymorphism | | |
